# The human RNA-binding protein RBFA promotes the maturation of the mitochondrial ribosome

**DOI:** 10.1042/BCJ20170256

**Published:** 2017-06-13

**Authors:** Agata Rozanska, Ricarda Richter-Dennerlein, Joanna Rorbach, Fei Gao, Richard J. Lewis, Zofia M. Chrzanowska-Lightowlers, Robert N. Lightowlers

**Affiliations:** 1 Institute for Cell and Molecular Bioscience, Newcastle University, Medical School, Framlington Place, Newcastle upon Tyne NE2 4HH, U.K.; 2 The Wellcome Centre for Mitochondrial Research, Institute of Neuroscience, Newcastle University, Medical School, Framlington Place, Newcastle upon Tyne NE2 4HH, U.K.

**Keywords:** mitochondrial gene expression, mitochondrial ribosomes, mitoribosomal maturation, ribosomal RNA maturation, ribosome assembly

## Abstract

Accurate assembly and maturation of human mitochondrial ribosomes is essential for synthesis of the 13 polypeptides encoded by the mitochondrial genome. This process requires the correct integration of 80 proteins, 1 mt (mitochondrial)-tRNA and 2 mt-rRNA species, the latter being post-transcriptionally modified at many sites. Here, we report that human ribosome-binding factor A (RBFA) is a mitochondrial RNA-binding protein that exerts crucial roles in mitoribosome biogenesis. Unlike its bacterial orthologue, RBFA associates mainly with helices 44 and 45 of the 12S rRNA in the mitoribosomal small subunit to promote dimethylation of two highly conserved consecutive adenines. Characterization of RBFA-depleted cells indicates that this dimethylation is not a prerequisite for assembly of the small ribosomal subunit. However, the RBFA-facilitated modification is necessary for completing mt-rRNA maturation and regulating association of the small and large subunits to form a functional monosome implicating RBFA in the quality control of mitoribosome formation.

## Introduction

Assembly and maturation of a fully functional ribosome is a demanding but fundamental feature of cellular metabolism. Budding yeast, for example, can produce 2000 ribosomes per minute [1], reflecting the demands of protein synthesis. Across almost all characterized species, the ribosome is composed of 50 or more components that must be correctly assembled to generate a small (SSU) and a large (LSU) ribosomal subunit, each with the appropriate modifications made to the component RNA and polypeptides. Mammalian mitoribosomes are no exception but the process of translation is far less well characterized. In most eukaryotes, mitochondria contain their own genome (mtDNA) and synthesis of these mtDNA-encoded polypeptides takes place within the organelle. This process uses mitoribosomes that contain 80 protein components that are nuclear-encoded, translated in the cytosol and then imported into the organelle. Here, they associate with the mitochondrially encoded rRNAs that are much reduced compared with their cytosolic counterparts and with one mtDNA-encoded tRNA that has become integral to the mt-LSU structure [2,3]. Assembly and maturation processes vary in different systems. Yeast cytosolic ribosomes, for example, require over 350 assembly factors, many of which are responsible for splicing and maturing the rRNA [4,5]. In comparison, eubacterial ribosome biogenesis appears to require relatively few factors, with GTPases being well represented among the 20 or so proteins that are known to be required [6,7]. Mitochondrial ribosomes differ between species [3,8–11] but for mammalian mitoribosome assembly, as with the eubacterial system, the number of key assembly factors is likely to be few [12–17]. One approach to identify candidate mitoribosome assembly factors has been by their similarity to bacterial factors. One such protein, ribosome-binding factor A, RbfA, is necessary for processing the 5′-terminus of the bacterial 17S rRNA precursor, to form the mature 16S rRNA component of the SSU [18–21]. This cleavage event appears to require the correct folding of helix (h) 1 of the rRNA [22], a claim supported by the crystal structure of RbfA alone, and by the cryoEM structure of the RbfA/*Thermus thermophilus* SSU complex [22]. The latter shows RbfA binding to the SSU neck region with its C-terminus close to h1 of the mature 16S rRNA. A plant orthologue of RbfA, RBF1, has recently been identified, but it is targeted exclusively to chloroplasts [23]. Interestingly, despite both chloro-ribosomes and mitoribosomes having bacterial origins, no mitochondrial RBFA orthologue has been identified in these plants, or indeed in the yeast *Saccharomyces cerevisiae*.

The 12S and 16S rRNA components of the human mt-SSU and mt-LSU are encoded by mtDNA [24]. These rRNA species are matured from a larger polycistronic RNA transcript [25]. Neither contains intronic sequences, nor do they require editing, or removal of nucleotides at either the 3′- or 5′-terminus [26]. Importantly, since neither rRNA requires any further processing after excision from the polycistronic unit, there is no apparent need for an RbfA orthologue in human mitochondria. However, a ‘ribosome-binding factor A (putative)’ protein appears in many databases. Despite this annotation as an orthologue, human RBFA bears little significant amino acid similarity to the *Escherichia coli* protein. Here, we present data that show RBFA is found in human mitochondria and does indeed play a role, albeit a different role from that in eubacteria, in rRNA maturation and ribosome assembly.

## Experimental procedures

### Cell culture and siRNA transfection

All cell types were grown in DMEM (Sigma D6429) supplemented with 10% FCS, 1× NEAA and 50 µg/ml uridine. Wild-type FLP-IN TRex 293 cells (Invitrogen: HEK293) were grown with 10 µg/ml Blasticidin^S^, and FLP-IN TRex 293 transfected lines with inducible expression of FLAG-tagged genes of interest were routinely treated with 100 µg/ml Hygromycin^B^. The transfected lines used in the present study were generated as described in ref. [14], except for RBFA that was created *de novo* using primers detailed in Supplementary Information. Expression of FLAG-tagged proteins was induced with 1 µg/ml tetracycline or 1 ng/ml doxycycline for RBFA-FLAG. Cells were cultured in humidified 5% CO_2_ at 37°C.

All custom and control non-targeting (OR-0030-NEG05) siRNA duplexes were from Eurogentec. Sequences of custom-synthesized siRNA sequences are given in Supplementary Information. HEK293T cell lines were reverse-transfected using Lipofectamine RNAiMAX (Invitrogen), Opti-MEM + Glutamax (Gibco) and a final siRNA concentration of 33 nM (NT, ERAL1) or 50 nM (si-RBFA 2), unless otherwise specified. Cells were re-transfected if required.

### Isokinetic sucrose gradient analysis

Cell (700 µg) or mitochondrial (300 µg) lysate or immunoprecipitated eluate (eluted by 3× FLAG peptide as per the Sigma FLAG IP protocol) was separated through linear sucrose gradients [10–30% (v/v) in 50 mM Tris–HCl (pH 7.2), 10 mM MgOAC, 40 mM NH_4_Cl, 0.1 M KCl, 1 mM PMSF and 50 μg/ml chloramphenicol; Beckman OptimaTLX bench ultracentrifuge, TLS55 rotor, 100 kg, 135 min, 4°C]. Fractions were collected and analyzed by western blot as described below or by silver staining as outlined in ref. [14].

### Cell lysate, mitochondrial, mitoplast preparation, SDS–PAGE and western blotting

Lysate was prepared by homogenization of cells on ice in 50 mM Tris–HCl (pH 7.4), 150 mM NaCl, 10 mM MgCl_2_, 1 mM EDTA, 1% (v/v) Triton X-100, 1× Roche protease inhibitor cocktail and 1 mM PMSF. Aggregates were removed by centrifugation (400 ***g***, 10 min at 4°C). Mitochondria were isolated by differential centrifugation and proteinase K-treated (4 μg/100 μg protein) in isolation buffer [10 mM Tris–HCl (pH 7.4), 0.6 M mannitol, 1 mM EGTA and 0.1% BSA] lacking BSA, on ice for 30 min, followed by the addition of 5 mM PMSF. Washed mitochondria were resuspended in isolation buffer and, where necessary, were solubilized with 1% (v/v) Triton X-100 or used to prepare mitoplasts, which required incubation on ice, in Tris–HCl (pH 7.4) in the presence of proteinase K, inactivated with 5 mM PMSF after 30 min. Mitoplasts were washed twice with isolation buffer containing 1 mM PMSF and RNase inhibitor. For western blot analysis, samples (50 µg) were separated by SDS–PAGE, transferred to Immobilon-P PVDF membrane (Millipore) and probed with relevant antibodies as follows: anti-RBFA polyclonal antibody was custom-synthesized by Eurogentec from human RBFA protein overexpressed in *E. coli* and purified following standard procedures: ERAL1 (11478-1-AP), MRPS18B (16139-1-AP), MRPS25 (15277-AP), ICT1/mL62 (10403-1-AP) ProteinTech Group; DAP3 (ab11928), L3 (ab39268), L12 (ab58334) Abcam; FLAG (F1804), β-actin (A1978) Sigma; S6 Ribosomal Protein (2317S) Cell Signalling; Porin (A31855) Molecular Probes; SDHA (MS204) Mitosciences; NDUFB8 (A31857), COX2 (A6404) Invitrogen.

### Immunoprecipitation of FLAG-tagged proteins

Immunoprecipitations via the FLAG moiety of tagged mL62, mS27, ERAL1 and RBFA were performed with the FLAG IP Kit (Sigma) as per the Sigma protocol with a minor buffer modification as described in ref. [14]. Sigma lysis and wash buffers were adjusted with Roche EDTA-free Protease Inhibitor Cocktail, 1 mM PMSF, 10 mM MgCl_2_ and 3 µl SUPERase In™ RNase Inhibitor (Ambion)/500 µl of buffer. For western blot analysis, co-immunoprecipitants were released from the resin with Laemlli sample buffer. RNA was extracted from the resin as per the supplier's protocol (TRIzol Invitrogen).

### Primer extension assay

Primer extension assays based on ref. [27] determined the relative amounts of methyl modification on 12S rRNA bases *A*_936_, *A*_937_ of h45. Primer (5′-GGTTCGTCCAAGTG-3′) was [γ^32^P]-ATP-labelled and purified on Illustra MicroSpin G-25 columns (GE Healthcare). Labelled primer was annealed to 4 µg of total RNA or 800 ng extracted after FLAG-mediated immunoprecipitation of tagged proteins. Unmethylated *in vitro* synthesized RNA acted as a negative control in parallel reverse transcription reactions containing MMLV Reverse Transcriptase (48 U Promega) and dNTP mix lacking dGTP (each at 40 µM final concentration). The reactions were incubated at 37°C for 45 min and quenched with loading buffer [80% (v/v) formamide, 1 mM EDTA, 0.1% (w/v) BPB and 0.1% (w/v) XCFF] including a primer alone control. Samples (3–5 µl) were separated on 10% polyacrylamide/8 M urea sequencing gels in 1× TBE buffer at 50 W. Signals were detected with the Typhoon FLA9000 and ImageQuant software (Molecular Dynamics, GE Healthcare). The amount of modified RNA was quantified as a percentage of the total extended primer (stop m^6^_2_A/[stop m^6^_2_A + fully extended] × 100).

### *In vivo* mitochondrial protein synthesis

Analysis of mitochondrial protein synthesis in cultured cells was performed as described previously [28]. After the addition of emetine, cells were pulsed with [^35^S]met/cys for 15 min. Samples (30–50 μg) were separated by 15% SDS–PAGE. Signals were visualized as above and gels were subsequently stained with Coomassie blue to confirm equal loading.

### Cross-linking immunoprecipitation

Cross-linking immunoprecipitation (CLIP) assays were as described in Ule et al. [29]. Briefly, cells or isolated mitoplasts were UV-irradiated on ice, harvested and lysed in Sigma FLAG lysis buffer adjusted with 0.1% SDS (v/v), Roche EDTA-free Protease Inhibitor Cocktail and Promega RNaseIn. Specific RNP complexes were immunoprecipitated. RNA species bound to the protein of interest were dephosphorylated, ligated to the 3′-RNA linker and end-labelled with γ^32^P. Protein–RNA complexes were resolved on SDS–PAGE, transferred to nitrocellulose (BA-85 Whatman) and subjected to autoradiography. Appropriately sized RNP complexes were excised and proteinase K-treated. Following ligation of a 5′ RNA linker RNA, CLIP tags were amplified by RT-PCR, and then cloned, sequenced and analyzed as described in ref. [14], or IonTorrent-sequenced as described in ref. [30].

## Results

### Bacterial and human RBFA proteins are structurally related

**Figure 1.**
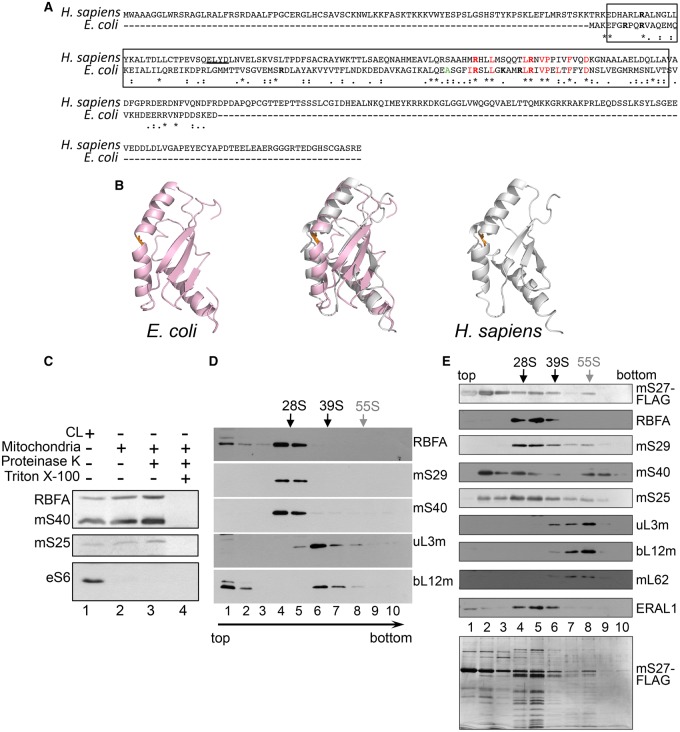
The human orthologue of bacterial RbfA associates with the mitoribosomal SSU. (**A**) Amino acid alignment (ClustalW) of RBFA from human (NP_079081.2) and *E. coli* (P0A7G2) shows identities as (*), high level of similarity by (:) and lower levels by (.). Boxed region indicates the predicted position of ‘ribosome-binding’ and RNA-binding ‘KH’ domains. The basic residues (in bold) Arg7, Arg10, Arg45, Arg80, Lys85 and Arg90 in *E. coli* RbfA are implicated in RNA binding [21]. Ala75 (orange) forms an inter-helical kink shown in **B**. A conserved sequence signature (I**R**XXLXXXXXL**R**XVPXLXFXXD) is located in the C-terminal region of *E. coli* RbfA. Human RBFA shares most but not all of these characteristics. (**B**) NMR-derived structures of *E. coli* RbfA (pink; PDB 1KKG, [21]) and the corresponding region of human RBFA that excludes the N- and C-terminal extensions (*H. sapiens*, silver; PDB 2E7G, [53]) are depicted individually and superposed using Coot [31]. Both exhibit type II KH domain folds (three helices and three β-strands). Human RBFA has an additional short helix, underlined in the panel **A** alignment. The inter-helical kink, formed in *E. coli* by Ala75 (1KKG) and Ser159 (2E7G) in human, is shown in orange. (**C**) Lysate (50 µg, lane 1) and mitochondria (10 µg, lanes 2–4) were prepared from HEK293 cells. Isolated mitochondria were treated with proteinase K in the absence (lane 3) or presence (lane 4) of 1% Triton X-100. Western blots detected RBFA, mitochondrial matrix markers (mitoribosomal subunits mS40 and mS25) and a cytosolic marker (cytosolic ribosomal subunit eS6). (**D**) HEK293 cell lysate (700 μg) was separated through a 10–30% sucrose gradient. Fractions were analyzed by western blot, using antibodies against mt-SSU (mS29 and mS40) and mt-LSU (uL3m and bL12m) components. The positions of the mt-SSU (28S), mt-LSU (39S) and the monosome (55S) are indicated. RBFA distribution was determined using antibodies against the endogenous protein. (**E**) FLAG-tagged mS27 was expressed, immunoprecipitated and the immunoprecipitate separated by sucrose gradient centrifugation. Fractions were subjected to western blot to detect the monosome, the mt-SSU, and its assembly intermediates (fractions 2–3). A silver-stained gel of the fractions is shown below.

Human RBFA comprises 343 amino acids, with a predicted molecular mass of 38 kDa (GeneCards, ref. seq. NP_079081.2). This is in contrast with eubacterial RbfA proteins that are less than half the size, ranging from 13 to 15 kDa; the *E. coli* protein (strain K12; P0A7G2), for example, comprises only 133 amino acids (Interpro, http://www.ebi.ac.uk/interpro/entry/IPR023799). BLAST could not align the human and *E. coli* protein sequences. ClustalW stated 15% identity and 16% similarity when human RBFA was aligned with the 133 amino acids of the *E. coli* protein (Figure 1A). RbfA homologues are described as containing ‘ribosome-binding’ and RNA-binding ‘KH’ domains, which in the human protein are predicted to lie in the central portion (amino acids 88–198, InterPro, http://www.ebi.ac.uk/interpro/protein/Q8N0V3; Figure 1A, boxed region). Even over these two relatively conserved domains, there is little identity, although some sequence similarity exists. Despite this, and excluding the N- and C-terminal extensions present in human RBFA, there is clear structural similarity over the KH domains in the two proteins, as both display a type II KH domain fold of three helices and three β-strands (Figure 1B). For both the *E. coli* and the *Homo sapiens* RbfA NMR structural studies, only ensembles have been deposited rather than a single lowest energy conformer, and, consequently, we arbitrarily chose to compare model 1 from each of the submitted structures. Using Coot [31], the RMSD (root-mean-square deviation) of the C alphas was determined to be 2.6 Å. This was based on a global secondary structure superposition of 88 amino acids from the 108 found in structure 1KKG (*E. coli*; pink) and the 129 residues in structure 2EKG (*H. sapiens*; silver). For clarity, the disordered residues at the N- and C-termini (Gly79 to Gly85 and Ala189 and Gly207) in 2E7G have been omitted. This existing structural similarity between the human and bacterial proteins is consistent with the former having retained an RNA-binding function. Human RBFA has, however, substantial extensions, both N- and C-terminal to the RNA-binding/KH domains, which may have evolved functions additional to or different from those of the bacterial protein.

### RBFA associates with the mt-SSU

GeneCards describes human RBFA as ‘putatively’ mitochondrial, consistent with the N-terminal extension driving its mitochondrial localization, but predictions are mixed (TargetP 1.1, 94.6% confidence of mitochondrial localization *cf* PSORTII at 43.5%). To clarify this issue, cell lysates and mitochondrial fractions were analyzed by western blot (Figure 1C, lanes 1 and 2, respectively). Isolated mitochondria were proteinase K-shaved in the absence (lane 3) or presence of Triton X-100 (lane 4). The latter lyses the organelles and, when added together with proteinase K, confirms susceptibility of intramitochondrial proteins to the protease. The pattern of mitochondrial enrichment for RBFA corresponds to that of known mitoribosomal proteins (mS25 and mS40) and contrasts with the S6 cytosolic ribosomal protein, confirming RBFA to be a mitochondrial protein.

To determine whether RBFA associates with the small (mito)ribosomal subunit (mt-SSU) as found for its bacterial orthologue, HEK293 cell lysates were subjected to isokinetic sucrose gradient fractionation. The distribution of mt-SSU and mt-LSU protein components was visualized by western blot, and RBFA was found to co-migrate with mt-SSU proteins (Figure 1D; mS29 and mS40). To distinguish more accurately which mitoribosomal components RBFA associates with, a FLAG-tagged mitoribosomal protein (mS27) was expressed, by which both mt-SSU and 55S particles could be immunoprecipitated. Following 3 days of mS27-FLAG expression, mitochondria were isolated and mS27 immunoprecipitated. The immunoprecipitated complexes were competitively eluted from the beads and subjected to sucrose gradient separation. RBFA could be detected predominantly with the mt-SSU, confirming that the substantial majority of RBFA was mt-SSU-associated (Figure 1E).

### RBFA is an RNA-binding protein

**Figure 2.**
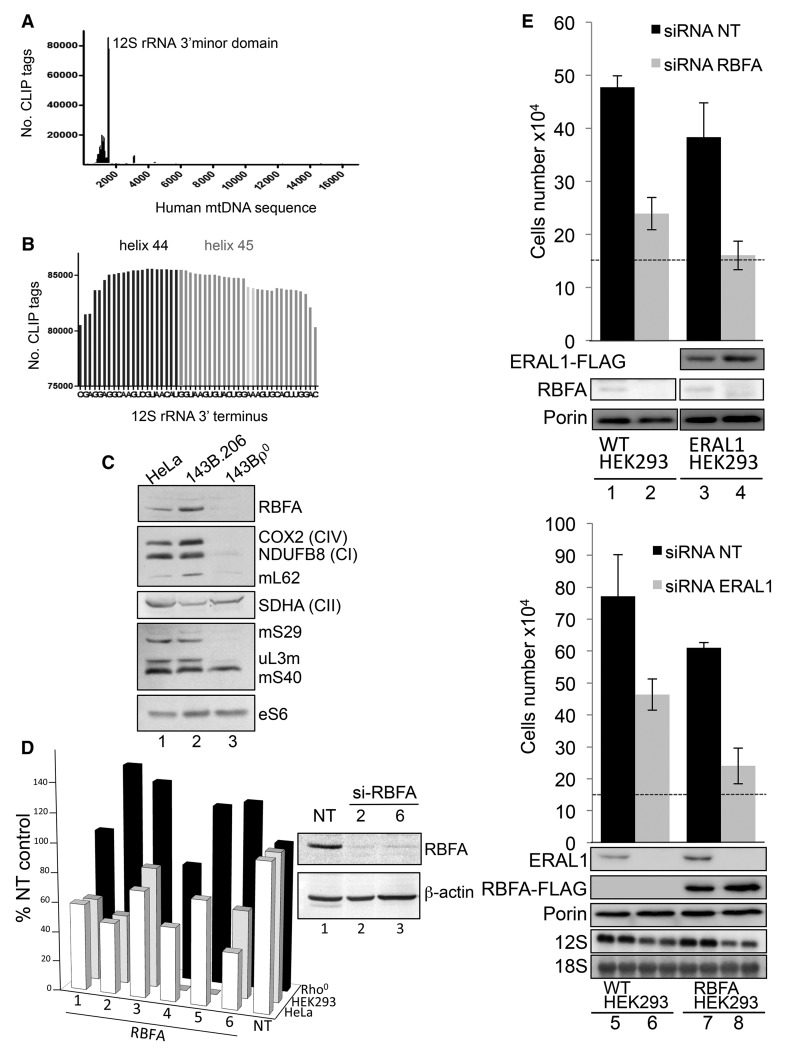
RBFA binds to helix 45 of 12S rRNA but has a distinct function from that of ERAL1. (**A**) The graph represents the human mtDNA sequence with locations and numbers of the CLIP tags indicated. Tags were generated as described in the Experimental Procedures. (**B**) The location of the greatest number of CLIP tags from three independent experiments is depicted spanning helix 45 and protruding into helix 44. The two dimethylated adenines in helix 45 are indicated in light grey. The terminal C-residue represents the near 3′-terminus of the 12S mt-rRNA, corresponding to nt1597 of mtDNA. (**C**) Lysates (30 μg) from HeLa, 143B.206 parental and Rho^0^ cells were analyzed by western blot to compare the relative expression levels of RBFA, components of the OXPHOS complexes (COX2, NDUFB8 and SDHA) and members of the mitoribosome (mS29, mS40, mL62 and uL3m,). Cytosolic RP-S6 is also shown. (**D**) Cell growth of each cell line was determined after 3 days treatment of six RBFA-targeted siRNAs (33 nM) (lanes 1–6) compared with control (NT lane 7). Inset: western blot of cell lysates (25 µg) after treatment with RBFA siRNA 2 and 6 to assess the level of depletion with β-actin as the loading control. (**E**) Top panel: HEK293 cells were grown for 72 h in the presence of either si-NT (lanes 1 and 3) or si-RBFA (lanes 2 and 4). ERAL1-FLAG expression was induced 4 h after siRNA transfection (lanes 3 and 4). Lower panel: HEK293 cells were grown for 72 h in the presence of either si-NT (lanes 5 and 7) or si-ERAL1 (lanes 6 and 8). RBFA-FLAG expression was induced 4 h after siRNA transfection (lanes 7 and 8). All siRNAs were used at 33 nM. Final cell numbers are plotted, and initial numbers are indicated by dashed lines. Western blots (50 µg of cell extracts) confirmed depletion and correct expression using antibodies against RBFA, ERAL1, FLAG or porin as a control. Westerns are representative of experimental triplicates.

KH domains denote RNA-binding activity, and this domain has been thoroughly characterized for bacterial RbfA and the related plant protein, RBF1 [18–23]. In these organisms, maturation of the SSU rRNA requires multiple cleavages at the 5′-terminus, but this is not the case for human mt-rRNA. In human mitochondria, transcription units containing the mt-rRNA sequences are processed to release the individual 12S and 16S species without the need for further cleavage at either terminus. Therefore, even if the RNA-binding capacity and the association with the SSU were retained, the function of RBFA cannot be completely conserved. To determine if the interaction between RBFA and the mt-SSU seen in sucrose gradients (Figure 1D) was mediated by the ribosomal RNA and, if so, to assess the precise RNA-binding spectrum of RBFA under physiological conditions, CLIP was employed both on intact cells growing on tissue culture plates and on isolated mitochondria. Cross-linking was followed by lysis, and affinity purified antibody against RBFA was used to immunoprecipitate the endogenous protein together with any cross-linked RNA species. Bound RNA was extracted, used to generate a cDNA library and sequenced using IonTorrent. RBFA was found to bind almost exclusively to the 12S rRNA, in four independent locations, with the vast majority of protected fragments mapping to the 3′-terminal region (Figure 2A,B and Supplementary Figure S1). RBFA has, therefore, clearly retained the capacity to bind RNA. Moreover, as with the bacterial protein, RBFA physiologically binds the ribosomal RNA from the SSU, albeit near the 3′- but not 5′-terminus.

### Loss of RBFA affects cell homeostasis

If the primary function of RBFA is to act on mitochondrially encoded rRNA, it is reasonable to assume that it may not be stable in Rho^0^ cells that lack mtDNA and thus cannot assemble mitoribosomes. A similar lack of stability has been well described for many other components of the mitoribosome and factors involved in mtDNA expression in Rho^0^ cells. To test this hypothesis, lysates were prepared from human cell lines: HeLa, osteosarcoma 143B.206 parental and 143B Rho^0^. Comparing 143B Rho^0^ cells (Figure 2C, lane 3) with controls (lanes 1–2), expression of cytosolic proteins was at similar levels, mt-encoded proteins were absent and steady-state levels of RBFA were at the limit of detection, implicating a role in mitochondrial gene expression. To investigate this further, the effect of RBFA depletion using six independent siRNA duplexes was assessed in the same three cell lines alongside a non-targeting (NT) control siRNA. In both control cell lines, a significant decrease in growth rate was observed (Figure 2D, graph), recapitulating the defect reported for RbfA-deleted bacteria [20,32]. The lack of a growth defect in Rho^0^ cells reinforced the potential role of RBFA in mitochondrial gene expression. Two siRNAs were selected for continued investigations in HEK293 cells (Figure 2D, inset). After 6 days of RBFA depletion, the mtDNA copy number was unaffected (data not shown), and a minimal increase in the steady-state level of some mt-RNAs and a mild increase from 1.7% apoptotic cells in controls to 7.4% could be detected (Supplementary Figure S2A,B).

### ERAL1 and RBFA are not interchangeable in human cell lines

Our CLIP data showed that RBFA-bound helices 44 and 45 of the 12S rRNA, covering the site where the GTPase and 12S mt-rRNA chaperone, ERAL1, has also been shown to bind [14,33]. Furthermore, ERAL1 was the only mitochondrial gene identified (www.genecards.org) to share a similar RNA-binding KH domain. The bacterial orthologue of ERAL1, Era, also binds in the vicinity of the 3′-end of the SSU rRNA [34,35]. It is also involved in bacterial ribosome biogenesis [36], and interestingly, in bacteria, Era overexpression can partially suppress *RbfA* deletion mutant defects arising in 16S rRNA maturation and ribosome assembly [19,37]. Since these data implied that the two proteins have overlapping functions in bacteria, we aimed to determine if the same functional redundancy exists in human cells. We, therefore, depleted RBFA with concomitant overexpression of ERAL1 in HEK293 cells. Unlike the suppression seen in bacteria, RBFA-depleted HEK293 cells grew equally poorly with or without the expression of ERAL1 (Figure 2E, upper panel). The reciprocal experiment did not restore either the control levels of growth or the steady-state levels of 12S rRNA, which became decreased when ERAL1 was absent (Figure 2E, lower panel).

### Loss of RBFA does not cause any immediate measurable mitochondrial dysfunction

**Figure 3.**
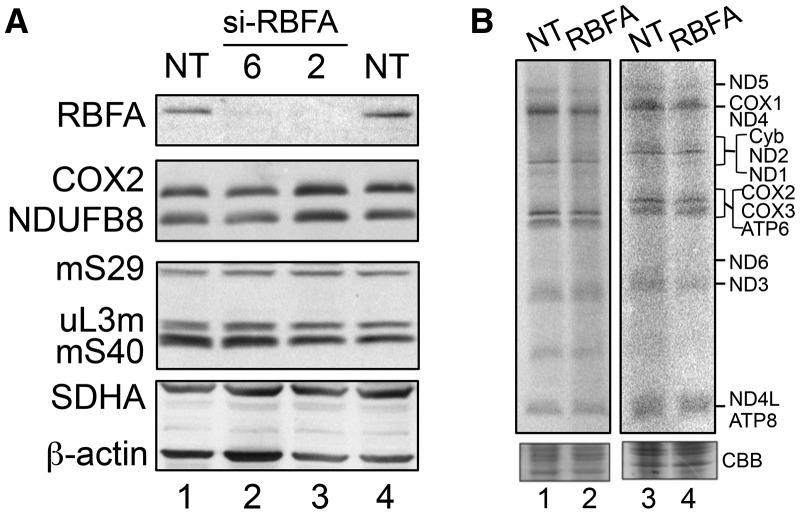
Depletion of RBFA has no appreciable effect on mitochondrial protein synthesis. (**A**) HEK293 cells were treated with RBFA (lanes 2 and 3) or non-targeting (NT, lanes 1 and 4) siRNA for 3 days and extracts (25 µg) were subjected to western blotting to compare OXPHOS (COX2, NDUFB8 and SDHA) or mitoribosomal (mS40, mS29 and uL3m) protein levels. (**B**) Following similar siRNA treatment (NT or RBFA-2) for 3 (lanes 1 and 2) or 6 (lanes 3 and 4) days, cells were metabolically labelled (^35^S-met/cys) and extracts (3 days—30 µg; 6 days—50 µg) separated by 15% denaturing PAGE. Migration of the 13 mtDNA-encoded polypeptides is indicated and loading confirmed by Coomassie blue (CBB) staining.

As the lack of RBFA is clearly deleterious for the cell, we aimed to determine the molecular pathogenesis. The association of RBFA with mitoribosomes infers that the growth defect may be due to a problem in synthesizing mtDNA-encoded proteins. Cells depleted of RBFA demonstrated no significant decrease in steady-state levels of individual OXPHOS components when assessed by western blotting (Figure 3A). This was consistent with metabolic labelling of the mitochondrially encoded proteins that was also unaffected on short-term depletion of RBFA (Figure 3B). The standard parameters associated with mitochondrial dysfunction were then assessed. No significant differences, relative to non-target (NT) siRNA controls, were observed for mitochondrial membrane potential, or superoxide levels, while a minimal increase in mitochondrial mass was apparent (Supplementary Figure S2C). Intriguingly, a similar lack of mitochondrial dysfunction has been noted previously for cells depleted of ERAL1 [14]. Using an siRNA approach, that study also identified a defect in cell growth in ERAL1-depleted cells. Under those conditions, little *de novo* mt-SSU assembly was observed; however, the remaining intact mt-SSU was sufficient to maintain normal levels of mitochondrial protein synthesis and, thus, steady-state levels of OXPHOS components. Both cases, depletion of either RBFA or ERAL1, resulted in profound growth defects before any effect on gross mitochondrial function could be measured.

### Loss of RBFA reduces the modification of 12S rRNA

**Figure 4.**
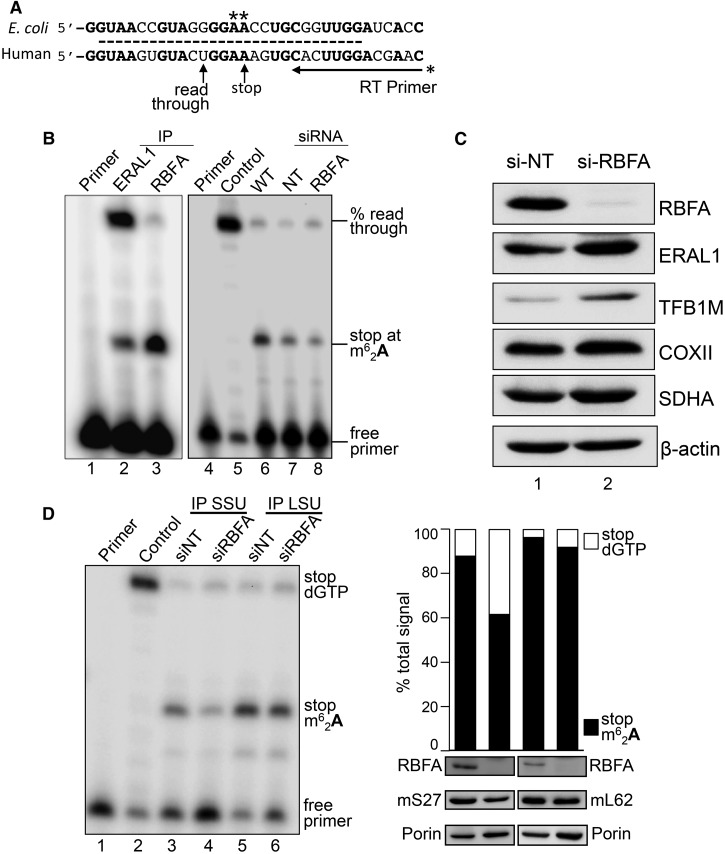
Depletion of RBFA causes a decrease in 12S rRNA modification. (**A**) Schematic presenting sequence conservation of helix 45 (dashed line) and the primer extension assay used to measure the modification levels at adenine residues *A*_936_/*A*_937_ in human 12S rRNA. If the modification is present, the primer extends four residues, and in its absence the primer extends a further five residues. (**B**) To determine the modification status of 12S rRNA bound to ERAL1-FLAG (lane 2, 3-day induction) or RBFA-FLAG (lane 3, 3 days), these proteins were immunoprecipitated from HEK293 cells and the bound RNA was extracted. Samples were subjected to primer extension and denaturing PAGE. (Right panel) Cells were depleted of ERAL1 (3 days) followed by 4 days of siRNA treatment; si-NT (lane 7) or si-RBFA (lane 8). RNA was extracted and primer extension performed. Primer alone (lane 1, 4), extension on unmethylated template (lane 5) and wild-type cells (lane 6) were controls performed in parallel. (**C**) Western blot (50 µg of cell extracts) determined the steady-state levels of ERAL1, TFB1M and COXII in cells depleted of RBFA. SDHA and β-actin were used as loading controls. (**D**) HEK293 cells were grown for 5 days in the presence of NT or RBFA siRNA with induction of mS27-FLAG (IP SSU) or mL62-FLAG (IP LSU) on day 3. (Left panel) Cell extracts were subjected to immunoprecipitation and bound 12S rRNA was assessed by primer extension alongside controls (primer alone, lane 1; unmethylated control, lane 2), and then visualized and quantified as described. (Right panel) Densitometric analysis is presented for lanes 3–6 from the left panel (stop m^6^_2_A_937_ = arrested at dimethylation; stop dGTP = full read-through). Western blots of the initial extract (50 µg) are shown probed for RBFA, mS27/mL62 via FLAG and porin.

Our data indicate that the interaction of RbfA with both the SSU and the rRNA observed in bacteria is also seen in human mitochondria. Their corresponding rRNA species, however, differ, as mammalian mt-rRNA is much reduced in size compared with its bacterial counterpart [38]. This loss of nucleotides is not random but represents removal or shortening of specific peripheral helices [38]. Notable is the retention of the terminal helix, h45, which remarkably, considering the lack of primary sequence conservation over the entire rRNA, shares identity with 18 of the 26 nucleotides (Figure 4A) and is a major RNA-binding region identified for RBFA (Figure 2A,B and Supplementary Figure S1). Of these identical residues, two consecutive adenines (*A*_936_, *A*_937_; numbering reflects position within human 12S mt-rRNA) are situated in the tetraloop capping the apex of h45. Not only is their position highly conserved but so is their modification, as each is dimethylated [27,39–41]. Loss of this modification in bacteria has been shown to compromise SSU/LSU and SSU/IF3 interactions, leading to defects in translation. The latter manifest as decreased fidelity at both ribosomal A and P sites, elevated initiation from non-AUG codons and increased stop codon read-through and frameshifting [42,43]. Therefore, to determine the status of the dimethylation modification of mitochondrial 12S rRNA, we utilized a primer extension assay. Under the assay conditions used, primer extension was arrested at modified base *A*_937_ [44] or, in the absence of modification, terminated to position U_933_ as no dGTP was in the reaction (Figure 4A). The assay revealed modest variations in the modification levels in several human cell lines (73–93%; Supplementary Figure S3). Since both RBFA and ERAL1 bind h45, we first sought to clarify the order of their interaction with 12S rRNA and the modification status at each stage. Parallel immunoprecipitations of endogenous RBFA and ERAL1-FLAG were performed and bound RNA was extracted for analysis. Strikingly, only 4% (*n* = 2) of the RBFA-bound 12S rRNA was unmethylated at h45 in contrast with 74% (*n* = 2) in the ERAL1 immunoprecipitation (Figure 4B, lane 3 *cf* 2). These data identified that ERAL1 associates with 12S rRNA prior to RBFA during mitoribosome biogenesis. We then proceeded to analyze the consequences of RBFA depletion on 12S mt-rRNA modification in HEK293 cells. As mitoribosomes are relatively stable and the substantial majority of 12S rRNA is already modified at steady state, it was important to enrich for newly synthesized (and unmodified) 12S rRNA to maximize any measurable effects of RBFA depletion on 12S modification. Thus, to enrich for newly synthesized 12S rRNA, the level of mature and partially assembled mt-SSU was first reduced by depletion of ERAL1 (the 12S mt-rRNA chaperone, [14]) from all cells for 3 days. Repletion of ERAL1 was then undertaken to stabilize nascent 12S concomitant to RBFA depletion or si-NT control (4 days), and total RNA was extracted and subjected to primer extension. The amount of unmodified 12S rRNA increased substantially on depletion of RBFA from the control average of 13.7 ± 1.4%, *n* = 3 (Figure 4B, lane 7) to 34.7 ± 1.2%, *n* = 4 (lane 8; *P* < 0.0001). These data indicate a role for RBFA in promoting 12S modification, an important maturation step of mt-SSU rRNA. It was unclear at the molecular level how RBFA may mediate this modification. However, preliminary microarray analyses on RBFA-depleted cells showed a modest 1.9-fold increase in TFB1M, the methyltransferase reported to be responsible for the modification [45], and 1.7-fold increase in ERAL1 (data not shown). These findings were supported at the protein level, where enrichment of signal was noted for TFB1M and ERAL1 in HEK293 lysate from si-RBFA cells compared with si-NT control (Figure 4C). Taken together with the primer extension assay performed on 12S rRNA immunoprecipitated via ERAL1-FLAG (Figure 4B), this suggests that loss of RBFA may trigger less efficient compensatory ways of promoting dimethylation.

As RBFA depletion increased the proportion of unmodified h45, we next sought to determine whether this unmodified rRNA species was incorporated into the mt-SSU, or whether correct 12S adenine dimethylation represents a quality control step in full assembly of the mt-SSU. To address this, the mt-SSU was immunoprecipitated from HEK293 cells via a FLAG-tagged component, mS27, after RBFA depletion (5.5 days). The proportion of unmodified h45 in the immunoprecipitated mt-SSU was 38.4 ± 4.2%, *n* = 3. In contrast, only 12 ± 6.3%, *n* = 3, was unmodified in the non-targeting (NT) siRNA control immunoprecipitation (Figure 4D, lanes 3 and 4; *P* = 0.0011). This demonstrates that unmodified 12S rRNA can be incorporated into assembled mt-SSU. Does the lack of 12S modification preclude mt-LSU interaction? To determine if unmodified h45 was represented in the intact monosome, RBFA was depleted prior to immunoprecipitation of mt-LSU via FLAG-tagged mL62. The 12S rRNA signal derived from the mt-LSU immunoprecipitation therefore represented assembled monosome. In contrast with the mt-SSU IP, h45 modification of the 12S rRNA was apparent on 96 ± 4% (*n* = 3) in the non-targeted control and 92 ± 6% (*n* = 3) of the RBFA-depleted cells (Figure 4D, lanes 5 and 6). These data suggest that correct modification is an important quality control step, permitting only modified mt-SSU to associate with the mt-LSU, thus ensuring that assembled monosomes will be translationally efficient. This observation can also explain how *de novo* synthesis of mitochondrial proteins could remain unaffected after 3 days of RBFA depletion (Figure 3), a phenomenon that was previously detected upon ERAL1 depletion [14]. Despite the significant effect on assembly and maturation of mitoribosomes following the loss of these key factors, nascent synthesis is still supported by the recycling of fully matured mt-SSU that remains during the depletion period.

## Discussion

Loss of rRNA modifications in bacteria has been known for some time to cause reduced growth rates [46] and may also reduce ribosome recycling [47]. The characterization and roles of rRNA modifications in the human mitoribosome are less well established, although data on *A*_936_ and *A*_937_ have been published [27,44,48]. The involvement of RBFA in this modification process, however, has not previously been documented. Our data show that RBFA has a role in promoting dimethylation of the 12S rRNA in the human mitoribosome.

*How might RBFA promote dimethylation of the tandem adenines in helix h45?* In humans, both ERAL1 and RBFA bind helix 45 of the 12S rRNA. Prior to RBFA binding, ERAL1 must first chaperone 12S rRNA during biogenesis of the mt-SSU. Our immunoprecipitation data indicated that, at this stage, the majority of ERAL1-bound RNA is unmethylated, whereas the RBFA-associated 12S rRNA was almost exclusively methylated. CryoEM data of RbfA and Era complexed with the 30S SSU from *T. thermophilus* reveal occupancy at different positions but in close proximity [22]. Our mass spectrometric analysis of proteins co-immunoprecipitating with RBFA did not detect significant levels of ERAL1 (unpublished observation), inferring that dual occupancy on human 12S rRNA is unlikely, particularly as the vast majority of CLIP-protected RNA fragments associated with the two proteins were found to be similar. Detailed NMR and X-ray crystallographic analyses have determined the structure of RbfA in various bacterial species as well as the structurally conserved KH/ribosome-binding domain from human RBFA [21,49] (PDB ID 2KZF; 2E7G). Another study showed that, in line with its role in the cleavage of the 5′-terminus of the eubacterial rRNA, RbfA is found at the neck of the SSU, where helix 1 of the 16S rRNA is located, at a junction of all four domains of the SSU, and where h1 and h44/45 are in relatively close proximity. Indeed, the binding of bacterial RbfA causes a structural rearrangement displacing h45 together with the abutting section of h44 by ∼25 Å [22]. RbfA binding also causes significant alterations in the location of the RNA-dependent intersubunit bridges, B2a and B3, precluding any association with the 50S large subunit [22]. Furthermore, RbfA binding overlaps with the position of the anticodon stem loops of both A- and P-site tRNAs, preventing the ingress of the fmet-tRNA^met^, mRNA binding and therefore translation initiation. In the human mitochondrial ribosome, not only is the h44 and h45 structural unit conserved, but despite many differences between the intersubunit bridges of 70S and 55S ribosomes, both B2a and B3 are also present [38,50,51]. This is consistent with our various analyses indicating that RBFA binds the mt-SSU and associates poorly with the 55S monosome.

The cryoEM data of *T. thermophilus* SSU in complex with RbfA clearly show that it binds in the cleft, essentially burying itself inside the SSU and is found behind helices 44/45 rather than on the surface of the intersubunit face [22]. This is consistent with the specificity of binding that we see with our CLIP data and might predict that RBFA pushes helix 45 outwards, exposing the tandem adenines, making them more accessible for the methyltransferase. Thus, although not directly responsible for the methyltransferase activity, RBFA appears to facilitate more efficient modification that may occur although with lower efficiency in the presence of ERAL1.

This may also explain why the proportion of unmodified h45 was not more marked when RBFA was depleted. It is important to note that at least for the bacterial SSU rRNA *in vitro*, modification is not absolutely essential for ribosomal function, as reconstitution with unmodified 16S rRNA produces a functional subunit, albeit one that translates with a drastically reduced efficiency [45]. In contrast, we show that despite RBFA depletion in intact cells, 55S particles retained near-maximal levels of *A*_936_, *A*_937_ dimethylation compared with reduced levels in total mt-SSU. These data imply that, in live cells, only fully modified mt-SSU is licensed to become part of a functional monosome and that RBFA plays a role in the quality control of ribosome biogenesis in human mitochondria.

Depletion of RBFA caused a dramatic cellular phenotype prior to any detectable loss of mitochondrial translation or OXPHOS activity. Although loss of RBFA affected the level of mt-SSU maturation, normal levels of protein synthesis were maintained by promiscuous use of the remaining fully matured mt-SSU, as was previously noted following ERAL1 depletion [14]. What, therefore, causes the growth defect in these cells? We are currently unable to explain this phenomenon but made similar observations upon depletion of several other components necessary for mitochondrial translation [14,52] (unpublished observation). We are currently exploring this intriguing signalling process.

**Figure 5.**
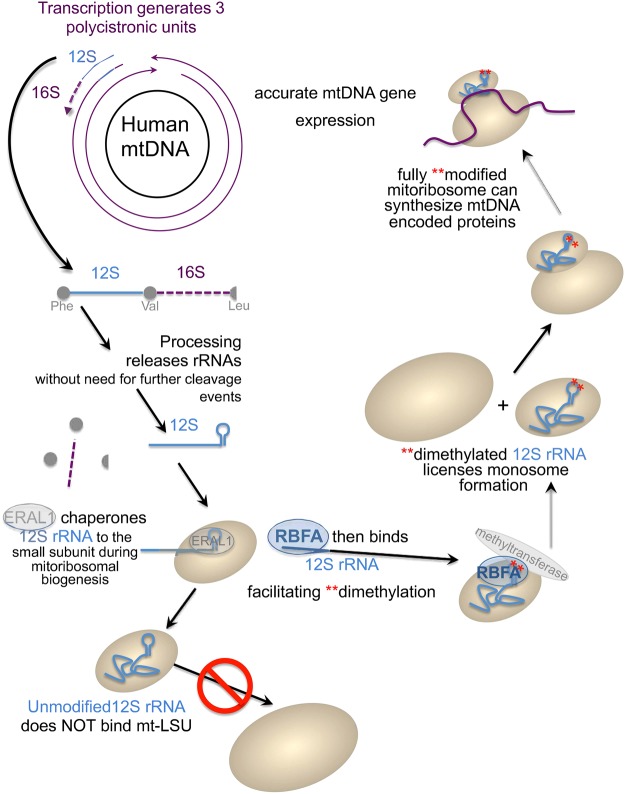
A schematic depicting the role that human RBFA plays in the maturation process of the 12S rRNA that is incorporated into the small subunit of the mitoribosome.

In conclusion, our data show that RBFA is important for 12S rRNA maturation as it plays a role in facilitating the modification of helix 45. It is likely that helix 45 needs to present a specific structure to the methyltransferase for optimal dimethylation of *A*_936_, *A*_937_, and that the RBFA binding provides the most favourable conditions for the final stages of 12S rRNA maturation (Figure 5). Moreover, these data indicate that the cell carefully monitors this maturation step, as failure to correctly assemble and maintain the mitoribosome triggers cell growth defects in advance of OXPHOS dysfunction or other markers of mitochondrial distress.

## Abbreviations

BPB: bromophenol blue
CLIP: cross-linking immunoprecipitation
COXII: cytochrome c oxidase subunit 2
ERAL1: Era like 12S mitochondrial rRNA chaperone
LSU: large ribosomal subunit
mt: mitochondrial
NEAA: non-essential amino acids
OXPHOS: oxidative phosphorylation
PMSF: phenylmethane sulfonyl fluoride
RBFA: ribosome-binding factor A
RNP: ribonucleoprotein
rRNA: ribosomal RNA
SSU: small ribosomal subunit
SDHA: succinate dehydrogenase subunit A
TFB1M: mitochondrial transcription factor B1
XCFF: xylene cyanol FF
